# Why do we think? The dynamics of spontaneous thought reveal its functions

**DOI:** 10.1093/pnasnexus/pgae230

**Published:** 2024-06-12

**Authors:** Judith N Mildner, Diana I Tamir

**Affiliations:** Department of Psychology, Princeton University, Princeton, NJ 08540, USA; Department of Psychology, Princeton University, Princeton, NJ 08540, USA

**Keywords:** spontaneous thought, cognition, natural language processing, memory, goal pursuit

## Abstract

Spontaneous thought—mind wandering, daydreaming, and creative ideation—makes up most of everyday cognition. Is this idle thought, or does it serve an adaptive function? We test two hypotheses about the functions of spontaneous thought: First, spontaneous thought improves memory efficiency. Under this hypothesis, spontaneous thought should prioritize detailed, vivid episodic simulations. Second, spontaneous thought helps us achieve our goals. Under this hypothesis, spontaneous thought should prioritize content relevant to ongoing goal pursuits, or current concerns. We use natural language processing and machine learning to quantify the dynamics of thought in a large sample (*N* = 3,359) of think aloud data. Results suggest that spontaneous thought both supports memory optimization and keeps us focused on current concerns.

**Significance Statement**

Spontaneous thought—the mind wandering, daydreaming, and creative ideation our mind defaults to—is a cornerstone of everyday cognition. Why do we devote so much time to spontaneous thought? Two longstanding hypotheses about the function of spontaneous thought propose it may serve to (i) optimize memory and (ii) keep the mind focused on ongoing goal pursuits. Spontaneous thought is notoriously difficult to capture empirically. Here, we use big data and natural language processing to test these hypotheses. Results suggest that the dynamics of spontaneous thought facilitate both memory optimization and a focus on goals. Additionally, we illustrate how natural language processing can uncover the dynamics of spontaneous thought to address fundamental questions about the nature of thought.

## Introduction

People spend most of their mental lives thinking. Given the chance, the mind defaults to wandering through unbidden imagination, memories, ideas, and daydreams (1–6). These thoughts are extremely common: Almost everyone daydreams at least once per day (7) and mind wandering makes up an estimated 25–47% of waking thought (8–12). Why do we spend so much time and cognitive resources thinking?

This freely flowing stream of thought is also known as spontaneous thought. We define spontaneous thought as thought that is relatively free from deliberate constraints (13, 14), aligning with William James' early descriptions of the stream of thought (15). This mode of thought has also been labeled stream of consciousness (16), resting state thought (17), idle thought (18), and unconstrained thought (19), and includes specific types of thought, such as task-unrelated thought (20), stimulus-independent thought (1), and mind wandering (13, 21), as well as related constructs like creative ideation (13) and rumination (22). It does *not* include highly deliberative or task-focused thoughts that are subject to strong cognitive control. Spontaneous thought, particularly mind wandering, is sometimes considered a failure of attention or executive function. However, spontaneous thought may, instead, serve an adaptive purpose that merits the vast amounts of cognitive resources and time spent on these thoughts.

Here, we quantify the dynamics of spontaneous thought to gain insight into its function. Specifically, we investigate two hypotheses about the function of spontaneous thought (14). These hypotheses are informed by empirical and theoretical work on spontaneous thought and related constructs, as well as parallel investigations into constructs such as daydreaming, mind wandering, involuntary memory, and memory replay. First, spontaneous thought may facilitate encoding new memories and semantic abstraction (23–28). This hypothesis derives from research showing that the neural dynamics of spontaneous thought resemble those of memory replay, a process optimized for memory consolidation (13, 29–31). Second, spontaneous thought may prepare people to act on their current concerns (32–34). This hypothesis derives from empirical research showing that goal-relevant content is prioritized during mind wandering and daydreaming (33). We discuss each of these possibilities in turn.

### Memory optimization hypothesis

Memory replay is the spontaneous reactivation of memory traces in the hippocampus. In humans, memory replay occurs during sleep and waking rest and helps to consolidate memory for new experiences (24, 25, 27–29, 35–40). Memory replay is effective at consolidating both episodic and semantic information, in part because of its dynamics. During memory replay, memory traces are not reactivated in their original order and context (41–43). Instead, memory traces can be reactivated in novel sequences. Novel sequences help decorrelate episodic memory patterns, reducing competition between similar memories (44). In these sequences, memories are reinstated multiple times in different contexts. This facilitates generalization into semantic knowledge (45). Thus, the dynamics of memory replay—where episodes are recalled in a somewhat random order and context—facilitates memory consolidation (46).

Spontaneous thought mirrors memory replay, suggesting that it, too, may facilitate memory consolidation. First, people default to engaging in spontaneous thought during rest, the same context in which people engage in memory replay (1, 47, 48). Second, the neural dynamics of spontaneous thought mirror the neural dynamics of memory replay (13, 49–51). Third, the content of spontaneous thought consists primarily of episodic events—up to 60% by some experience sampling estimates (12, 26). These episodic events include recollections of past episodes and recombinations of past episodes in the form of future or counterfactual simulation (21, 52–57).

Because of these parallels, prior theoretical work has proposed that spontaneous thought is an unconstrained memory process where memory traces are reactivated in novel and variable sequences (14, 26). If so, spontaneous thought's variability, repetition, and pseudo-random sequencing may optimize semantic and episodic memory consolidation—just like memory replay (26). That said, this hypothesis has yet to be tested. Do episodic replays during spontaneous thought play any role in memory consolidation, or does this reflect the idle wandering of an unconstrained mind? *If* this continuous replay and recombination of memories in the form of episodic simulation functions as memory replay, this suggests that a key function of spontaneous thought is to optimize memory.

To test this possibility, we track episodic detail in spontaneous thought. Why episodic detail? Prior work shows that episodic detail is necessary both to decorrelate episodic memories and to optimize semantic knowledge. While there are likely many other mechanisms to improve memory, here we use the link between these types of memory optimization and episodic detail as a proxy for memory optimization more generally. If memory optimization is a key function of spontaneous thought, then spontaneous thoughts with episodic details should be prioritized over more abstract or semantic thoughts. There is already some evidence that thought prioritizes episodic detail. Specifically, spontaneous thought typically centers on psychologically proximal content (e.g. recent experiences; (54, 58–60)), which contains more episodic detail than distal content (61–63). However, the possibility that thought dynamics prioritizes episodic detail has not been directly tested.

The overlap between spontaneous thought and memory replay suggests that the function of spontaneous thought may be to optimize memory (14, 26). Here, we test this possibility by developing novel automated analysis techniques to assess the trajectory of spontaneous thought in terms of episodic detail.

### Current concerns hypothesis

People often maintain many goals at one time. For example, I may be concurrently concerned with finishing the book on my nightstand, writing a lecture, and not catching a virus. If I was deliberately completing or planning for these goals, I would be engaging in deliberately constrained thought (13). If so, this would *not* be considered spontaneous thought. However, ongoing goals can also spontaneously pop up in one's thoughts. For example, though you are deliberately trying to read this article, your mind may be wandering to thoughts about your own unfinished books or lectures. In this case, the content of these thoughts would be relevant to current concerns, but the thoughts would be unfolding spontaneously, without deliberative constraints or cognitive control.

Much of the content of spontaneous thought indeed comprises ongoing goal pursuits or current concerns (33, 64, 65). On average, 47% of thoughts are goal-relevant (66). People regularly report goal-relevant thoughts during rest (67) and mind wandering in daily life (9, 68). In fact, the same current concerns that occur in everyday thoughts, also surface in free association (69). Additionally, the future is a prevalent theme in spontaneous thought (52, 54). Cues heighten the likelihood that spontaneous thoughts will gravitate toward personal goals or planning the future (8). Thoughts reflect goal-relevant cues about twice as often as irrelevant cues (70), with important goals that people are highly committed to, have high incentive value, and require imminent or immediate action most likely to influence thought (71). Reminding participants of their ongoing goals, for example by making a to-do list, increases such goal-related future thinking even further (72, 73). The prevalence of goal-relevant and future oriented thought led to a theory that mental content, both spontaneous and goal-directed (74), continuously jumps from one goal-related topic to the next (64).

This emphasis on current concerns may reflect a key function of spontaneous thought: help pursue future goals. This focus can be adaptive in multiple ways. First, thoughts about current concerns serve as an ongoing reminder of overarching goals when we cannot directly engage with them. This may prepare us to act on current concerns when the opportunity arises (34, 52, 75, 76). There is some empirical evidence for this: For instance, in one study, the extent to which people activated brain regions associated with thinking about the self during rest predicted subsequent performance on a self-referential processing task (77). Second, spontaneous thoughts may offer the mind time to develop creative solutions for goal-related problems (64), though empirical tests on the effect of idle thought on creativity have found mixed results (78).

Together, these findings suggest that spontaneous thought prepares us for goal-relevant action and problem-solving by focusing our minds on current concerns. If facilitating goal pursuit is a key function of spontaneous thought, then thoughts that address a current concern are more “valuable” than wholly undirected thoughts (64, 79). While prior studies have shown the prevalence of goal-related content in spontaneous thought, it is not yet clear if the dynamics of spontaneous thought maximize current concerns-related content. Here, we test this possibility by investigating the trajectory of spontaneous thought in terms of relevance to a topic of peak concern at the time of data collection: the COVID-19 pandemic.

### Quantifying the function of spontaneous thought

The current concerns and memory optimization hypotheses are not mutually exclusive. Consolidating new information can facilitate effective future goal pursuits. For instance, when people have time for idle rest after listening to an emotional disclosure, they show better memory for the disclosure *and* greater social support towards the speaker the next day (80). How can we determine whether either of these hypotheses captures the function of spontaneous thought?

In prior theoretical work, we posed that spontaneous thought is an unconstrained memory process, where the content of spontaneous thought consists of episodic simulation scaffolded by semantic memory. The mind traverses this memory structure based on drifting context representations that implement varying combinations of different types of associations (14). This conceptualization of spontaneous thought allows us to adapt computational methods and theories from research on memory to spontaneous thought.

Here, we demonstrate how these theoretical insights can be investigated in practice. Specifically, we leverage the parallel between the dynamics of spontaneous thought and semantic fluency to test the function of spontaneous thought. Spontaneous thought has a clustered structure, with thoughts exploring a particular topic until it is time to jump to a new topic (16). The timing of these jumps can reveal the function of thought (14). If spontaneous thought serves some function, thoughts should jump when the current topic no longer effectively contributes to this function. They should then land on a new topic that helps achieve this function.

This same logic—that jumps reveal function—derives from work on semantic fluency tasks. In these tasks, participants are asked to list as many items in a certain category as possible. Responses to these prompts have a clustered structure. For example, when asked to name as many animals as possible, people may first list different kinds of pets, then farm animals, then aquatic animals, and so on. The timing for the jumps reveals something about the goal of the task and can be modeled using a spatial foraging model. In spatial foraging and semantic fluency, the goal is to maximize the number of items retrieved over time. Items form clusters in semantic or physical space. It starts out easy to search within a cluster, so people do so. But at some point, finding new items in the cluster becomes harder. At that point, it's time to jump to a new one (81). Thus, the timing of the topic jumps is tied to the rate of retrieval: people switch clusters when it takes too long to find a new item in the current cluster (82). Here, we apply this logic about semantic search to test the function of spontaneous thought.

If spontaneous thought is designed to optimize memory storage and facilitate learning, we should see these goals revealed through the timing of topic jumps. Since recalling and recombining episodic detail can help decorrelate episodic events and facilitate abstraction, we focus on the amount of episodic detail in thought, as opposed to more general semantic thought. If memory optimization is a goal of spontaneous thought, then thought jumps should prioritize episodic detail. This should produce thought patterns where topic jumps occur when the level of episodic detail in a cluster dips too low. The first thought in a new topic should then contain a lot of episodic detail, with each subsequent thought decreasing in episodic detail until the next topic jump.

If spontaneous thought is designed to keep our minds focused on current concerns, then thought jumps should prioritize content relevant to ongoing goal pursuits. This would produce a pattern where topic jumps occur when the level of goal relevance in a cluster dips too low. The first thought in a new topic should be highly goal-relevant, with each subsequent thought decreasing in goal-relevance until the next topic jump.

The current study examines the function of spontaneous thought by analyzing the timing of topic jumps in a large dataset of data on spontaneous thought, spoken aloud, or typed. We first implement a novel automated pipeline, using natural language processing and machine learning to individuate thoughts and demarcate thought clusters. We then test the memory optimization hypothesis by examining if thought jumps prioritize episodic details and the current concerns hypothesis by examining if thought jumps prioritize goal relevance. These dynamics can elucidate why we spend so much time and energy engaging in spontaneous thought.

## Results

We collected 3,359 responses from 1,679 participants between April 2020 and May 2021. To measure the dynamics of spontaneous thought, participants completed a Think Aloud task, where they narrated their stream of thought in real-time. While think-aloud protocols were initially used to study thought processes during tasks (83), these methods can also be used without any external task to study spontaneous thought (16, 17). Here, we gave participants a choice between speaking into their microphone or typing their thoughts to maximize the accessibility of the study. Hence, we have both written and spoken “think aloud” data.

Prior work with think aloud data relied on human coders to individuate thoughts and demarcate thought clusters (16). Here, we used automated natural language processing tools to process the think aloud data. First, audio was transcribed into text using OpenAI's Whisper (84). Then, we split the text into individual units of thought by detecting independent clauses (85). Finally, we used a clustering algorithm to identify topic jumps (86).

We started by testing one hypothesized function of spontaneous thought: that it serves to optimize memory. To test this hypothesis, we used a pre-existing algorithm to quantify the level of episodic detail in each thought. This algorithm estimates each sentence's episodic detail (vs. nonepisodic detail) (87). It was originally developed to automate scoring of autobiographical memory interviews. It performs well compared to manual scoring in identifying episodic details. We applied the algorithm to quantify the proportion of episodic details in each thought. Mixed linear models predicted episodic detail from thought position, controlling for repeated measures. We report standardized coefficients for each model.

**Fig. 1.**
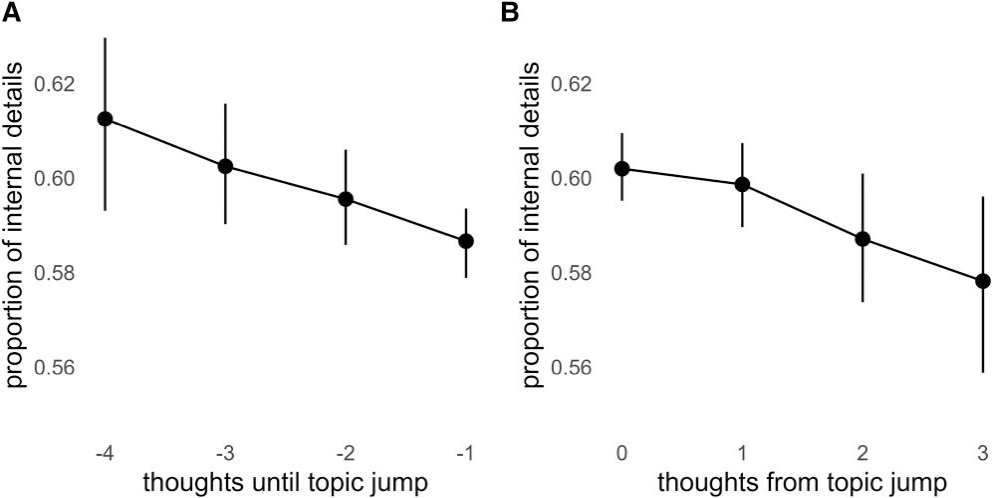
Spontaneous thought jumps show evidence of optimizing memory storage. Episodic detail A) decreases before a topic jump, B) increases at the topic jump, and then decreases again after the jump.

Results supported the hypothesis that the dynamics of spontaneous thought prioritize episodic detail (Fig. 1): there was a small but statistically significant effect of thought position on the amount of episodic detail in each thought, such that thoughts immediately after a topic jump had the highest level of episodic detail, and thoughts occurring later within a topic contained less episodic detail (*β* = −0.019, SE = 0.005, *P* < 0.001). When counting the position of thoughts until the next topic jump, we find the same pattern: the amount of episodic detail decreases leading up to a topic jump, with thoughts immediately preceding a topic jump showing the lowest level of episodic detail (*β* = −0.022, SE = 0.005, *P* < 0.001). Thus, the timing of topic jumps prioritizes episodic detail, suggesting that memory optimization is a goal of spontaneous thought.

We next tested the second hypothesized function of spontaneous thought: that it keeps us focused on current concerns. Since we did not collect data on participants' idiosyncratic current concerns, we chose a universal concern for people during the data collection period: the COVID-19 pandemic. To assess pandemic-relevance, we created a dictionary of pandemic-related words to count the proportion of pandemic-related words in each unit of thought (see Supplementary methods). Mixed linear models predicted pandemic-relevance from thought position, controlling for repeated measures.

**Fig. 2.**
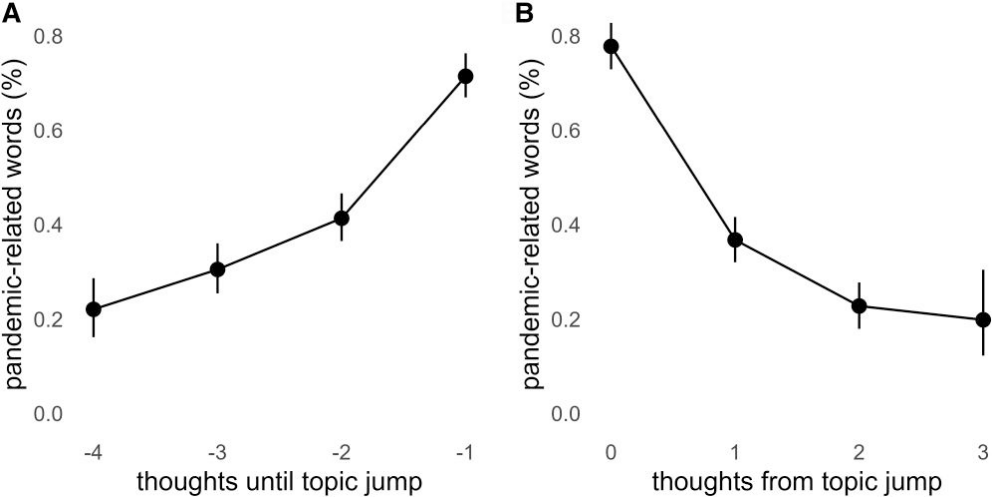
Spontaneous thought jumps show evidence of prioritizing goal-relevance. Current concerns-related content A) increases before a topic jump, then B) decreases after the jump.

Results partially supported the hypothesis that the dynamics of spontaneous thought prioritize current concerns (Fig. 2): there was a small but significant effect of thought position on the proportion of pandemic-related words in each thought, such that thoughts immediately after a topic jump had the highest level of pandemic-related words, and thoughts occurring later within a topic contained fewer pandemic-related words (*β* = −0.080, SE = 0.005, *P* < 0.001). For thoughts leading up to a topic jump, the observed pattern was the opposite of what was expected. Rather than decrease until the jump, thoughts immediately before the jump showed a gradual, significant increase in pandemic-related content (*β* = 0.064, SE = 0.005, *P* < 0.001). These results are inconsistent with the hypothesis that thought jumps occur to maximize a focus on current concerns. That said, the dynamics of spontaneous thought are strongly shaped by current concerns.

**Fig. 3.**
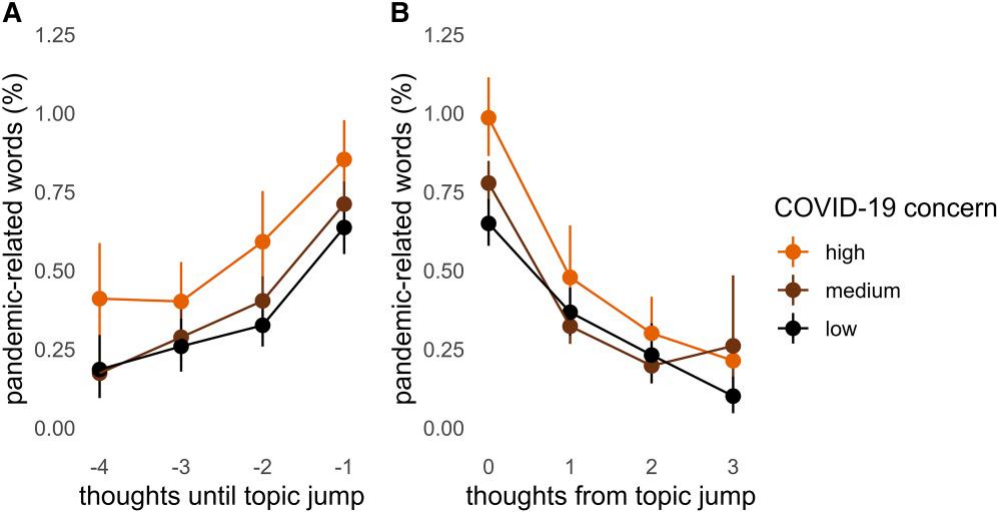
The more important the goal is to participants, the more it is prioritized in spontaneous thought. Participants with high concern about COVID-19 show the strongest evidence that current concerns-related content A) increases most before the topic jump and B) decreases most after the topic jump. The data were binned into low, medium, and high scoring thirds for visualization purposes. Statistical analyses used the raw scores of 0–6.

Participants in our sample varied in their level of concern about the COVID-19 pandemic. The more a participant was concerned about COVID-19, the more they showed the pattern of thought described above (Fig. 3). There was a significant interaction between thought position and COVID-19 concern after the topic jump, such that participants with higher COVID-19 concern showed a steeper decrease in pandemic-related content after the jump (*β* = −0.034, SE = 0.014, *P* = 0.015). There was no significant interaction between thought position and COVID-19 concern before the topic jump (*β* = 0.011, SE = 0.015, *P* = 0.469). We also found a significant main effect of COVID-19 concern, such that participants who were more concerned had more pandemic-related thoughts overall in both before (*β* = 0.039, SE = 0.011, *P* < 0.001) and after the topic jump (*β* = 0.043, SE = 0.008, *P* < 0.001). Finally, the main effects of thought position remained significant, with pandemic-related content decreasing after the topic jump (*β* = −0.049, SE = 0.013, *P* < 0.001) and increasing leading up to the topic jump (*β* = 0.042, SE = 0.013, *P* < 0.001). Thus, concern about COVID-19 amplified the effect of thought position on pandemic-related words, further supporting the hypothesis that current concerns play a role in shaping the dynamics of thought—albeit not in the same way as episodic detail.

## Discussion

This study tracked the dynamics of spontaneous thoughts to gain insight into its function, using natural language processing of think aloud data. We demonstrate how natural language processing and insights from computational memory research can be applied to think aloud data to uncover the function of spontaneous thought. We hypothesized that spontaneous thought functions to optimize memory and keep us primed to act on current concerns. We find support for both functions of spontaneous thought. When the current topic in thought no longer provides sufficient episodic detail for memory optimization, the mind jumps to a new topic with more available episodic detail. To prepare for goal-relevant action, this new topic will also be high in goal-relevance. This pattern of results suggests that the function of spontaneous thought is multifaceted: spontaneous thought prioritizes both episodic detail and current concerns.

These results join a growing body of work showing the importance of spontaneous thought for memory optimization. Activating episodic memory traces out of their original order can facilitate efficient memory storage and can help optimize semantic memory (26). Previous research has established that the content of spontaneous thought is largely episodic (12, 21, 52, 55, 57) and that spontaneous thought can facilitate memory consolidation (26, 27, 37). Here, we show that the dynamics of spontaneous thought indeed appear to be optimized to support this function. Future work could more closely examine the mechanisms of memory optimization during spontaneous thought by examining the dynamics of episodic detail in thought, how they relate to a specific recent experience, and contribute to subsequent recall.

We also find clear evidence that spontaneous thought is shaped by current concerns, albeit in an unexpected way. Specifically, instead of thought jumps occurring when current concerns dipped below a threshold, they occurred when current concerns were at a maximum. This pattern is most pronounced for participants who report the highest level of current concerns. Although current concerns clearly impact the dynamics of spontaneous thought, this pattern is inconsistent with our hypothesis. One possible reason why current concern content increases preceding a topic jump is that salient content may capture people's attention, encouraging them to think more about that content (32). That is, these stimuli may serve as attractor states, which can affect which portion of memory space that thoughts explore (14). Negatively valenced and anxiety-inducing topics, in particular, may encourage patterns of thought with this rumination-like dynamic, where thoughts repeatedly return to the same topic. That said, this study only measured the dynamics around a single, anxiety-inducing current concern: COVID-19. Thus, we cannot distinguish between the possibility that the dynamics of thought inherently prioritize all current concerns in this unexpected way, and the possibility that COVID-19 or features of this concern (e.g. anxiety) uniquely impact spontaneous thought in this way. We look forward to future work that takes a more individualized approach to more fully understand how the dynamics of spontaneous thought reflect a broader range of idiosyncratic current concerns, and individual differences in ruminative tendencies.

This study applied state-of-the-art tools in natural language processing and machine learning techniques to automate analyses of a large think-aloud dataset. Think-aloud data typically requires human coders to quantify the data. This approach would have been prohibitively labor-intensive and expensive, given the size of our dataset. Our automated approach for both sentence parsing and hierarchical clustering tools matched the reliability of human coders. That said, reliability remained moderate for both human and automated coders, suggesting that there is room for improvement in how we define individual units of thought and topic jumps—both conceptually and algorithmically. Additionally, the effect sizes were very small, likely in part because we used very short think aloud samples and natural language processing tools originally devised for longer, more structured text. Future work should endeavor to improve the accuracy and precision of these tools. Nevertheless, our automated tools allowed us to process a large amount of data with enough accuracy to capture small but practically significant variance in the dynamics of spontaneous thought, consistent with patterns predicted in prior theoretical work (14). Despite the imperfect methods, this study demonstrates that think aloud data combined with modern natural language processing tools can offer new insights into the contents and dynamics of spontaneous thought, even in largescale datasets.

Applying insights from semantic foraging models allows us to identify variables that describe the dynamics of thought. These variables can be used in more formal models. In spatial and semantic foraging, there is a computationally optimal patch-switching threshold that maximizes the rate of retrieval. Future work can apply this theoretically optimal threshold to episodic detail in spontaneous thought and examine how deviations from the optimal threshold affect thought patterns. For instance, if the threshold is too low, thoughts will rapidly jump from patch to patch, yielding disjointed, racing thoughts. On the other hand, if the threshold is set too high, thoughts will stay in one patch for a long time, leading to more repetitive, ruminative thoughts. The analysis techniques developed here highlight the value of quantifying this patch-switching threshold and open up the possibility of quantifying new metrics of the dynamics of spontaneous thought, including the semantic distance between thoughts and topics, thought speed, prevalence of topics, and affective dynamics. Tracking individual differences in these variables may explain individual differences in creative thought (88–90), or thought distortions in mental health disorders (13, 91–93), opening up future insights into otherwise elusive aspects of the dynamics of spontaneous thought.

Spontaneous thought makes up most of our daily cognition during wakefulness and dreaming. Why do we think so much? Natural language processing and insights from computational models of memory can make this elusive question empirically tractable. Here, we tested two hypotheses on why we devote so many cognitive resources to spontaneous thought: spontaneous thought helps optimize memory, and spontaneous thought keeps us focused on ongoing goal pursuits. Novel methods applied to a large dataset indicate that the dynamics of spontaneous thought support both functions. This shows that natural language processing can bridge the gap between big theoretical questions and empirical investigation of spontaneous thought. Additionally, results suggest that when we let our minds wander, thoughts are far from random or idle. Instead, they follow a pattern that optimizes memory function and keeps us aligned with our goals.

## Materials and methods

### Participants

We collected 3,359 responses from 1,679 participants (mean age = 39.8, range [19.0–79.0], 15.9% Asian/Asian American, 14.1% Black, 7.0% Latinx, 1.0% Native American, 65.7% White, 56.6% women, and 1.3% genderqueer/nonbinary/other) in three separate data collections between April 2020 and May 2021. Informed consent was obtained from all participants. The study was approved by the Princeton University Institutional Review Board. This study was conducted as part of a larger data collection effort. Participants completed up to 9 measures not included in this study, including surveys on the social environment and (social) well-being. Findings from other measures in the survey are available elsewhere (94).

#### Data collection 1

In the first data collection, participants were recruited through email lists at Princeton University and on social media. These participants volunteered their time and could participate as many times as they wanted, at least 5 days apart (*n* = 371; responses = 532).

#### Data collection 2

The second data collection occurred through the Center for Decision Research at the University of Chicago Booth School of Business. Participants received $4 each time they participated and were invited to participate twice, in April 2020 and May 2020 (*n* = 285; responses = 488).

#### Data collection 3

Participants in the final data collection were recruited through Prolific Academic (www.prolific.co) and received $4 for their participation. The first wave was collected in May 2020, and the same participants were invited again in June 2020 and April 2021 (*n* = 1023; total responses = 2311). In wave 2, 831 participants returned; in wave 3, 477 participants returned. These participants were a representative sample matched to U.S. census values for sex, age, and ethnicity.

#### Totals and exclusions

In every data collection, participants completed the study online on their own devices (mobile, tablet, or computer). For this study, we only included participants who spoke or wrote between 50 and 500 words in the Think Aloud task (see below; excluded *n* = 425). For those who provided audio recordings, we excluded participants whose audio consisted of at least 75% silence (*n* = 14) or who had poor audio quality operationalized as a speech-to-reverberation modulation energy ratio below 0.67 (*n* = 2). We then conducted manual inspections of all Think Aloud data and excluded low quality responses (*n* = 15), including non-English text, gibberish, copy-pasted instructions, cop-outs (e.g. writing “I’m not comfortable sharing my thoughts” over and over), and cases where the transcription picked up background noise from TVs or multiple speakers instead of the participant (*n* = 5). After exclusions, 1,524 participants provided 2,901 responses (Princeton: *n* = 331, responses = 506; Chicago: *n* = 248, responses = 414; Prolific: *n* = 945, responses = 2,016).

### Measuring spontaneous thought

To measure the dynamics of spontaneous thought, participants completed a Think Aloud task, as the first task presented during a more extensive data collection effort (94). During Think Aloud tasks, participants are asked to narrate their stream of thought in real-time. While think-aloud protocols were initially used to study thought processes during tasks (83), these methods can also be used without any external task to study spontaneous thought (16, 17).

In this study, participants were first told that they would record a short audio clip and received instructions on testing their microphones. After successful testing, participants were given the following task instructions:

“For 2 minutes, please report whatever you can on your stream of thought. Your report may include (but is not limited to) descriptions of: images, conversations, thoughts, sensations, feelings, memories of past experiences, thought or images of future experiences. Simply say whatever is going through your mind from moment to moment. Report your thoughts and feelings as they come to you. Try to talk continuously during the entire time. Don’t worry about grammar or complete sentences. If your thoughts or feelings recur over and over, simply say them over and over.

Remember that we are interested in your saying any and all thoughts and feelings that come to you no matter how silly or personal you think they may be.”

Next, participants saw a screen with a reminder to report their thoughts and feelings as they came to them and a record button. Upon pressing record, a progress bar appeared to indicate how much time was left in the task. The recording was stopped automatically after 2 min.

Participants could opt out of audio recording and type on their keyboards instead. Participants who opted out of audio recording saw a screen with similar instructions and a textbox in which they could type their thoughts. Instructions asked participants to type their thoughts and feelings as they came to them. Participants were also informed that only the latest portion of their response would be visible to them to allow them to focus on their current thoughts. The textbox only showed the most recent 100 characters. After the 2 min were up, a “next” button became active, and the participants could leave the task.

Out of 3,208 included responses, 819 were audio recordings, and 2,389 were written responses. On average, audio recordings contained 242.73 words (SD = 79.08), and written responses contained 98.15 words (SD = 49.74).

### Quantifying spontaneous thought dynamics

To quantify the dynamics of spontaneous thought, we first conducted preprocessing on the think-aloud audio recordings to transcribe the text and then split the text into individual units of thought. Finally, we grouped the individual thoughts into topics. Previous research has accomplished each of these steps using human coders to split think aloud data into individual thoughts and identify where topic jumps occur (16). We developed an analysis pipeline to replicate this procedure using automated natural language processing techniques to facilitate the processing of large datasets. In this pipeline, we build upon Sripada and Taxali's (2020) manual method by following their definitions of units of thought and topic jumps in order to identify their natural language equivalents.

### Identifying units of thought

All audio recordings were transcribed into text using Whisper (https://openai.com/research/whisper; (84)), an open source neural net for automated speech recognition. On a subset of the data, data collection 1, the audio was also transcribed using Temi (www.temi.com), an automated transcription service. Three research assistants edited the Temi transcripts for accuracy. We used these transcripts as a ground truth to assess the quality of the Whisper transcription and found that the word error rate was low (8%), confirming that the Whisper model provides accurate transcription in our data.

We then split each transcript and written think-aloud response into units of thought using the following automated process: We used a sentence parser to break each transcript into sentences and then identify coordinating conjunctions in each sentence (85). If a verb accompanied a coordinating conjunction, we considered this an independent clause. This method considers each independent clause a separate unit of thought.

To test the accuracy of this automated method, we compared the results of this method to the units identified by human coders. For this validation, human coders split 132 transcripts into units of thought following Sripada and Taxali's (16) definition of a unit of thought as the minimal unit of text that can stand on its own as a thought. In many cases, coders could use grammatical rules to identify units of thought by splitting the transcripts into sentences and then into independent clauses. In some instances, dependent clauses or partial sentences were separate units of thought if they expressed a complete and contentful thought. Two coders processed each transcript. To compare units across the human coders and automated method, we used the SegEval Python package to calculate inter-rater reliability (Fleiss' kappa) based on boundary similarity (95). Human coders showed moderate inter-rater reliability (Fleiss' kappa = 0.58). Human coders and the automated version showed comparable inter-rater reliability (Fleiss' kappa = 0.60). This suggests that the automated sentence parser performs comparably to human coders. While the automated method does not have as much nuance in identifying units of thought as human coders, it performed very well. Therefore, we used the automated solution to identify units of thought in our analyses.

### Identifying topic jumps

We defined a topic jump as any significant switch in time, place, and/or situation (16). Sripada and Taxali (16) showed that a hierarchical clustering algorithm could identify topic jumps with above-chance accuracy. Therefore, we used Scikit-learn in Python to implement hierarchical clustering (86) to identify which topic each unit of thought belonged to. On average, participants produced 10.82 thoughts (SD = 7.78) in 5.6 topics (SD = 4.31), with an average of 3.04 thoughts per topic (SD = 1.63).

To test the accuracy of this automated method, we compared the results of this method to the units identified by human coders on a subset of 91 transcripts. To compare topic jump coding, we again used the SegEval Python package to calculate inter-rater reliability (Fleiss' kappa) based on boundary similarity (95). Here, we find that the inter-rater reliability of human coders is 0.41; inter-rater reliability for the human coders and the automated solution was comparable, at 0.30. While the inter-rater reliability is fair to moderate, even for just the human raters, the automated solution does not perform notably worse than the human coders, suggesting that the automated solution is a viable method of replacing human coders to identify topic jumps.

### Quantifying episodic detail and current concerns content

If the function of spontaneous thought is to optimize memory efficiency, we expect the dynamics of spontaneous thought to prioritize episodic detail. This means that topic switches should occur once thoughts on the current topic contain insufficient episodic detail. To measure the amount of episodic detail in each thought, we use an automated scoring tool initially designed for autobiographical interview narratives (87). This software uses a fine-tuned language model to process sentences. Within each sentence, this software identifies the number of episodic details (i.e. internal details) and the number of nonepisodic details (i.e. external details). When applied to the units of thought in our dataset, this yields a proportion of episodic detail for each unit of thought (no. of episodic details/total no. of details). If spontaneous thought prioritizes episodic detail, we expect that the first thought after a topic switch has the most episodic detail, and the amount of episodic detail decreases with each subsequent thought.

Similarly, if the function of spontaneous thought is to prepare us to act according to current concerns, we expect the dynamics of thought to prioritize content related to current concerns. This means that topic switches should occur once thoughts on the current topic contain insufficient current concerns-related content. Since we did not collect data on participants' current concerns, we chose a universal concern for people during the data collection period: the COVID-19 pandemic. While this was likely a current concern for every participant in our sample, we also asked participants to indicate how concerned they were about COVID-19 on a 7-point scale. To assess pandemic-related content, we developed a dictionary of pandemic-related words. Using this dictionary, we counted the proportion of pandemic-related words in each unit of thought. If spontaneous thought prioritizes current concerns, we expect that the first thought after a topic switch would have the highest proportion of pandemic-related words, and the proportion of pandemic-related words would decrease with each subsequent thought. We expect this effect to be stronger for participants who are more concerned about COVID-19.

We tested these hypotheses using mixed linear models of episodic detail and pandemic-related words, respectively. These models contained a fixed effect for the position of thought within a topic, with 0 being the topic switch, 1 being the thought after the topic switch, and so on. The models also included random intercepts for data collection and participant. We did not include random slopes to reduce the complexity of the models and avoid overfitting and convergence issues. We added a second model to test how concern about COVID-19 affects current concern-related content. This model contained fixed effects for the position of thought within a topic, concern about COVID-19, and their interaction, and random intercepts for data collection and participant.

## Supplementary Material

pgae230_Supplementary_Data

## Data Availability

Data and code will be made available upon publication of this manuscript on the Open Science Framework (OSF): https://osf.io/k5whz/. Raw, unprocessed audio, and free response data will not be made publicly available, as these may contain identifiable information. Instead, we will make available all variables derived from audio, open-ended responses, and survey measures used in this manuscript.

## Preprint

This manuscript was posted as a preprint: https://doi.org/10.31234/osf.io/ufd4y.
