# Supplementary material for: Why do we think? The dynamics of spontaneous thought reveal its functions
Source: PNAS Nexus. 2024 Jun 12;3(6):pgae230. doi: 10.1093/pnasnexus/pgae230 (PMC11210302; doi:10.1093/pnasnexus/pgae230)
Supplement: pgae230_Supplementary_Data [file pgae230_supplementary_data.pdf]

PNAS nexus

# Supplementary Information for
Why do we think? The dynamics of spontaneous thought reveal its functions

Judith N. Mildner[1], Diana I. Tamir[1]

[1]Department of Psychology, Princeton University; Princeton, NJ, USA

*Judith N. Mildner.

**Email:** jmildner@princeton.edu

**This PDF file includes:**

Supplementary methods
Supplementary results
Figures S1 to S5
Tables S1 to S4

**Supplementary Methods**

**Pandemic-related words dictionary**

To measure the extent to which each thought was related to the pandemic, we used a custom LIWC dictionary. We developed this dictionary by identifying words related to the COVID-19 pandemic in news media and on social media. This dictionary is specific to pandemic-related words used in 2020 and 2021, and therefore does not include some common pandemic-related words that only became popular later, such as “booster.”

The words in the pandemic-related dictionary were (not case-sensitive):

*covid*
*covid19*
*covid-19*
*corona*
*coronavirus*
*virus*
*pandemic*
*lockdown*
*distanc**
*quarantin**
*isolati**
*mask**
*sanitizer*
*symptom**
*asymptomatic*
*cough**
*respirat**
*disease*
*illness*
*ventilator*
*ppe*
*self-isolat**
*non-essential*
*vaccin**
*vax**
*outbreak*
*spread**
*case**
*patient**
*delta*
*omicron*
*facemask*
*infect**
*restrict**

After these analyses were completed, LIWC updated their “health” category with some COVID-19 related words. The correlation between the pandemic dictionary and LIWC’s health category is $r$ = .33 ($p$ < 0.001), indicating some convergent validity for our dictionary.

**Supplementary Results**

For the purpose of cross-validation, we ran each of our analyses separately for each dataset. We found that the directionality of the results holds for every dataset. Episodic content decreases leading up to a topic jump and decreases after a topic jump, though the latter did not reach significance in the smaller datasets (Princeton & Chicago; see Table S1 for model statistics). Pandemic-related content increased leading up to the topic jump and decreased after the topic jump (see Table S2).

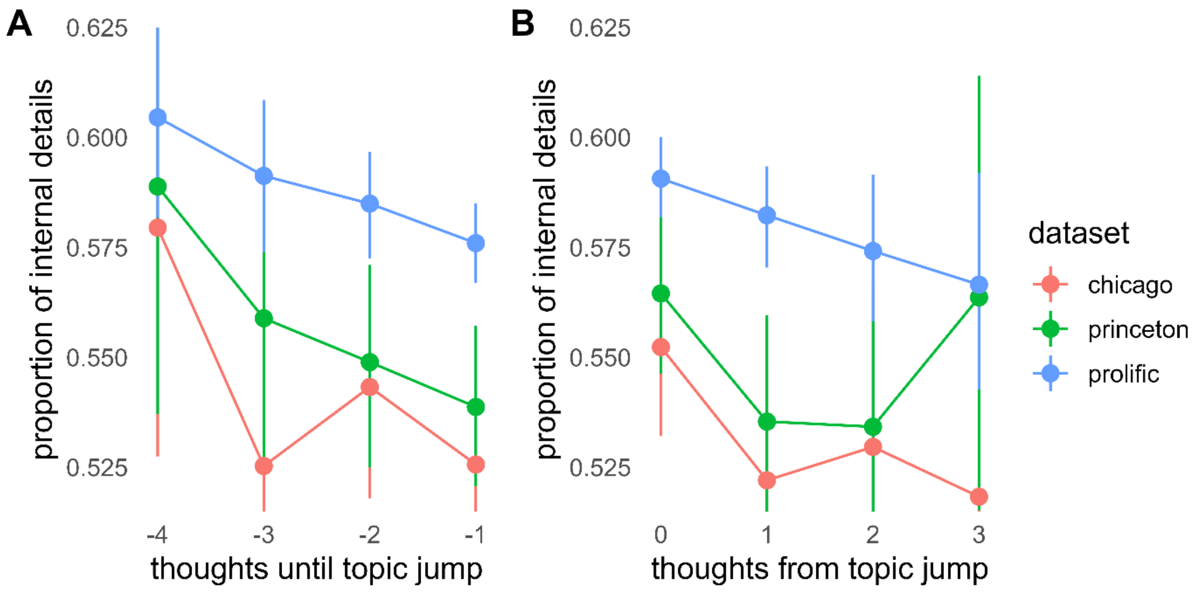


**Figure S1.** The pattern of results is consistent across datasets. Episodic detail (A) decreases before a topic jump, (B) increases at the topic jump, and then decreases again after the jump.

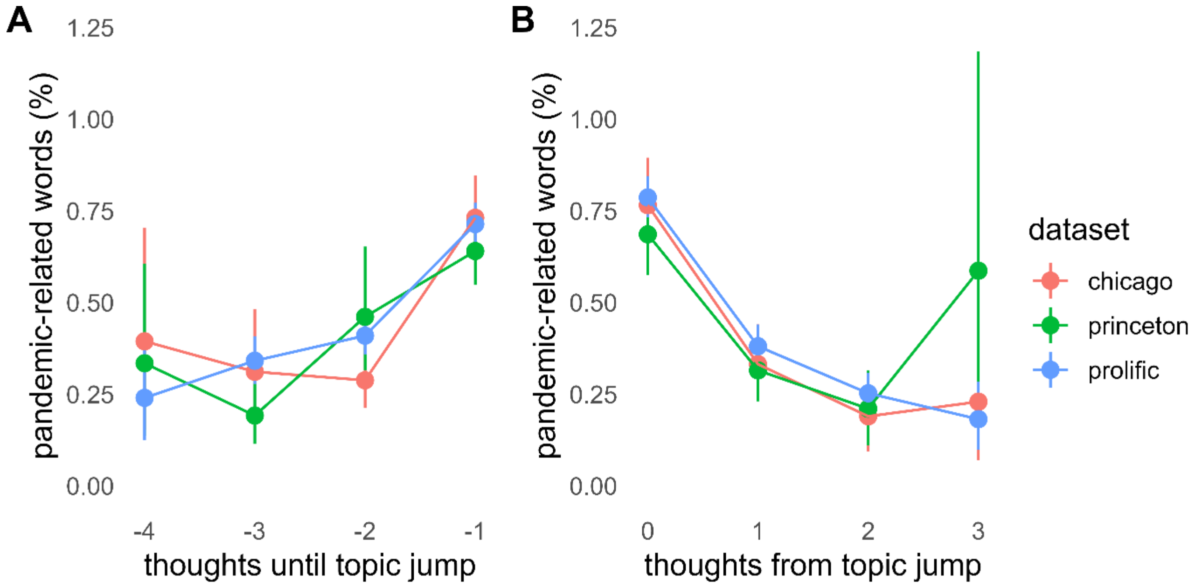


**Figure S2.** The pattern of results is consistent across datasets. Current concerns-related content (A) increases before a topic jump, then (B) decreases after the jump.

**Episodic detail before topic jump**

| | fixed effects | b | Std. Error | B | stdse | df | t value | p |
|---|---|---|---|---|---|---|---|---|
| **Chicago**<br>$N_{participants}$ = 271<br>$N_{observation}$ = 445 | Thoughts until jump | -0.014 | 0.005 | -0.038 | 0.014 | 5,121.8 | -2.706 | 0.007 |
| **Princeton**<br>$N_{participants}$ = 333<br>$N_{observation}$ = 467 | Thoughts until jump | -0.013 | 0.005 | -0.035 | 0.014 | 5,488.3 | -2.555 | 0.011 |
| **Prolific**<br>$N_{participants}$ = 974<br>$N_{observation}$ = 2127 | Thoughts until jump | -0.004 | 0.002 | -0.010 | 0.007 | 23,877.4 | -1.563 | 0.118 |

**Episodic detail after topic jump**

| | fixed effects | b | Std. Error | B | stdse | df | t value | p |
|---|---|---|---|---|---|---|---|---|
| **Chicago**<br>$N_{participants}$ = 271<br>$N_{observation}$ = 445 | Thoughts until jump | -0.007 | 0.005 | -0.021 | 0.014 | 5,122.5 | -1.469 | 0.142 |
| **Princeton**<br>$N_{participants}$ = 333<br>$N_{observation}$ = 467 | Thoughts after jump | -0.009 | 0.005 | -0.024 | 0.014 | 5,489.7 | -1.738 | 0.082 |
| **Prolific**<br>$N_{participants}$ = 974<br>$N_{observation}$ = 2127 | Thoughts until jump | -0.006 | 0.002 | -0.018 | 0.007 | 23,876.8 | -2.797 | 0.005 |

**Table S1.** Model coefficients for episodic detail for each dataset. Across datasets, episodic detail decreases leading up to a topic jump then increases on the topic jump and decreases after a topic jump.

**Pandemic-related words before topic jump**

| | fixed effects | b | Std. Error | B | stdse | df | t value | p |
|---|---|---|---|---|---|---|---|---|
| **Chicago** $N_{participants}$ = 271 $N_{observation}$ = 445 | Thoughts until jump | 0.121 | 0.026 | 0.067 | 0.014 | 4,924.686 | 4.729 | 0.000 |
| **Princeton** $N_{participants}$ = 333 $N_{observation}$ = 467 | Thoughts until jump | 0.108 | 0.028 | 0.053 | 0.014 | 5,245.355 | 3.879 | 0.000 |
| **Prolific** $N_{participants}$ = 974 $N_{observation}$ = 2127 | Thoughts until jump | 0.106 | 0.013 | 0.055 | 0.007 | 23,514.955 | 8.387 | 0.000 |

**Pandemic-related words after topic jump**

| | fixed effects | b | Std. Error | B | stdse | df | t value | p |
|---|---|---|---|---|---|---|---|---|
| **Chicago** $N_{participants}$ = 271 $N_{observation}$ = 445 | Thoughts until jump | -0.156 | 0.026 | -0.086 | 0.014 | 4,931.286 | -6.085 | 0.000 |
| **Princeton** $N_{participants}$ = 333 $N_{observation}$ = 467 | Thoughts after jump | -0.097 | 0.028 | -0.047 | 0.014 | 5,246.904 | -3.473 | 0.001 |
| **Prolific** $N_{participants}$ = 974 $N_{observation}$ = 2127 | Thoughts until jump | -0.156 | 0.013 | -0.080 | 0.007 | 23,508.700 | -12.313 | 0.000 |

**Table S2.** Model coefficients for current concerns for each dataset. Across datasets, pandemic-related content decreases leading up to a topic jump then increases on the topic jump and decreases after a topic jump.

**Audio vs written think aloud**

Participants were given the choice to write their thoughts rather than audio record if they did not have a working microphone or were uncomfortable recording. Most participants opted to write (73% of responses). There were some differences between written and spoken 'think aloud' data: audio responses contained more words (audio: *M* = 251.91, *SD* = 69.7; written *M* = 101.36, *SD* = 49.7) and more thoughts per response (audio *M* = 18.34, *SD* = 8.57; written *M* = 8.78, *SD* = 4.78; see Figure S3).

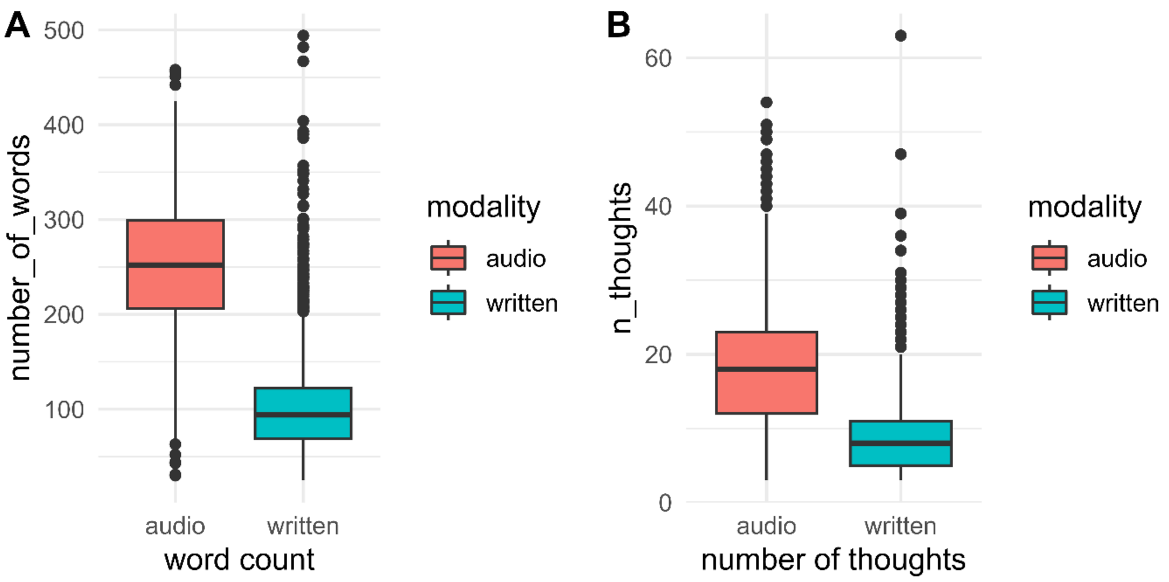


**Figure S3.** Audio responses contain more words and more thoughts than written responses.

When analyzed separately, audio responses and written responses show similar patterns of results. Episodic detail decreases before and after topic jumps, although this effect does not reach significance in the audio data (see Table S3 and Figure S4). Preprocessing on the audio data was relatively minimal compared to other datasets using the episodic detail scoring, which, combined with the lower sample size in audio data, may explain this lack of results. Pandemic-related content significantly increases leading up to a topic jump and decreases after the jump in both written and audio data (see Table S4 and Figure S5).

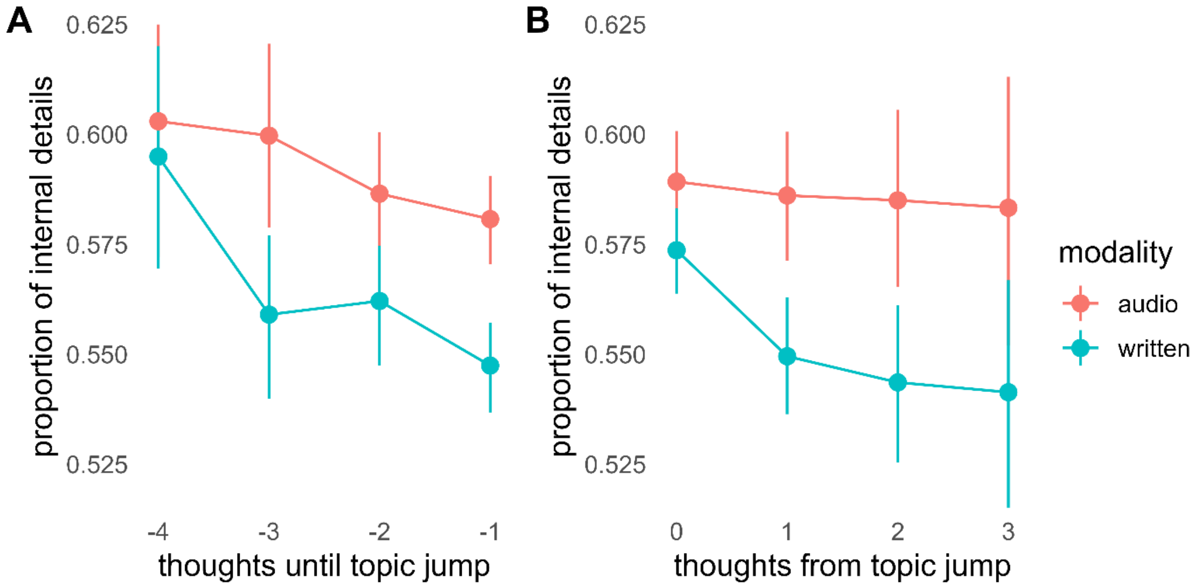


**Figure S4.** In written responses, episodic detail decreases before and after the topic jump. In audio responses, the pattern of results is similar but does not reach significance.

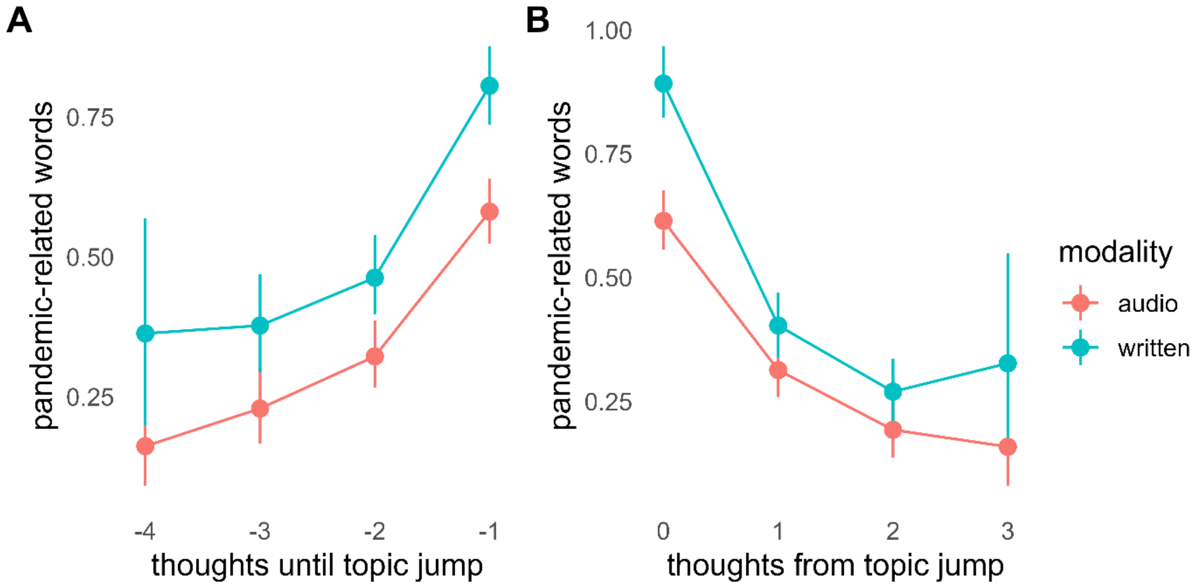


**Figure S5.** In both written and audio responses, pandemic-related content increases before the topic jump and decreases after the topic jump

| Episodic detail before topic jump | | | | | | | | |
|---|---|---|---|---|---|---|---|---|
| | fixed effects | b | Std. Error | B | stdse | df | t value | p |
| **Audio**<br>$N_{participants}$ = 563<br>$N_{observation}$ = 834 | Thoughts until jump | -0.004 | 0.003 | -0.012 | 0.008 | 15,060.673 | -1.421 | 0.155 |
| **Written**<br>$N_{participants}$ = 1251<br>$N_{observation}$ = 2207 | Thoughts until jump | -0.009 | 0.003 | -0.024 | 0.007 | 19,266.799 | -3.321 | 0.001 |
| **Episodic detail after topic jump** | | | | | | | | |
| | fixed effects | b | Std. Error | B | stdse | df | t value | p |
| **Audio**<br>$N_{participants}$ = 563<br>$N_{observation}$ = 834 | Thoughts after jump | -0.003 | 0.003 | -0.009 | 0.008 | 15,054.121 | -1.148 | 0.251 |
| **Written**<br>$N_{participants}$ = 1251 | (Intercept) | 0.546 | 0.021 | 0.000 | 0.000 | 2.143 | 26.253 | 0.001 |
| $N_{observation}$ = 2207 | Thoughts after jump | -0.009 | 0.003 | -0.026 | 0.007 | 19,275.037 | -3.643 | 0.000 |

**Table S3.** Model estimates for episodic detail by modality

| Pandemic-related words before topic jump | | | | | | | | |
|---|---|---|---|---|---|---|---|---|
| | fixed effects | b | Std. Error | B | stdse | df | t value | p |
| **Audio**<br>$N_{participants}$ = 563<br>$N_{observation}$ = 834 | Thoughts until jump | 0.093 | 0.013 | 0.057 | 0.008 | 14,76 0.318 | 6.983 | 0.000 |
| **Written**<br>$N_{participants}$ = 1251<br>$N_{observation}$ = 2207 | Thoughts until jump | 0.125 | 0.016 | 0.058 | 0.007 | 18,70 2.925 | 8.001 | 0.000 |

| Pandemic-related words after topic jump | | | | | | | | |
|---|---|---|---|---|---|---|---|---|
| | fixed effects | b | Std. Error | B | stdse | df | t value | p |
| **Audio**<br>$N_{participants}$ = 563<br>$N_{observation}$ = 834 | Thoughts until jump | -0.124 | 0.013 | -0.076 | 0.008 | 14,76 3.038 | -9.275 | 0.000 |
| **Written**<br>$N_{participants}$ = 1251<br>$N_{observation}$ = 2207 | Thoughts after jump | -0.169 | 0.016 | -0.079 | 0.007 | 18,69 6.561 | -10.871 | 0.000 |

**Table S4.** Model estimates for pandemic-related content by modality

**Validation of topic jumps**

To further verify that our algorithm identified real topic jumps, we analyzed a non-linguistic feature of the think aloud audio data: time. In other words, the time between thoughts should be larger for across topic switches than for thoughts within a topic. These temporal dynamics define typical semantic foraging task behavior as well, where there are longer inter item intervals when people recount an item from a new semantic cluster than within the same cluster.

To test this, we took our audio data (N= 811) and obtained estimated timestamps for the start and end of each word using OpenAI's Whisper model. We then mapped these timestamps to the start and end of each thought and compared the time in between thoughts at topic jumps and non-topic jumps. We tested if there was a difference in timing between these two types of thoughts jumps using a mixed linear model predicting time difference from topic jump (yes or no), with a random intercept for response. Indeed, we find that the time in between thoughts is significantly longer ($\beta$ = 0.042, *SE* = 0.008, $p < 0.001$) for topic switches (*M* = 0.82, *SD* = 1.50) than non-topic switches (*M* = 0.74; *SD* = 1.45). This offers convergent evidence that our method is indeed picking up on topic boundaries.
